# Automated radiogrammetry is a feasible method for measuring bone quality and bone maturation in severely disabled children

**DOI:** 10.1007/s00247-016-3548-4

**Published:** 2016-03-30

**Authors:** Sandra Mergler, Stella A. de Man, Annemieke M. Boot, Karen G. C. B. Bindels-de Heus, Wim A. R. Huijbers, Rick R. van Rijn, Corine Penning, Heleen M. Evenhuis

**Affiliations:** Department of General Practice and Intellectual Disability Medicine, University Medical Centre, Erasmus MC, Rotterdam, The Netherlands; Medical Department ASVZ, Care and Service Centre for People with Intellectual Disabilities, Sliedrecht, The Netherlands; Department of Paediatrics, Amphia Hospital, Breda, The Netherlands; Department of Paediatric Endocrinology, University Medical Centre Groningen, University of Groningen, Groningen, The Netherlands; Department of General Paediatrics, Sophia Children’s Hospital, University Medical Centre, Erasmus MC, Rotterdam, The Netherlands; Department of Paediatrics, Beatrix Hospital, Gorinchem, The Netherlands; Department of Radiology, Emma Children’s Hospital/Academic Medical Centre, Amsterdam, The Netherlands

**Keywords:** Automated radiogrammetry, Bone age, Bone maturation, Bone quality, Children, Disability, Radiography

## Abstract

**Background:**

Children with severe neurological impairment and intellectual disability are prone to low bone quality and fractures.

**Objective:**

We studied the feasibility of automated radiogrammetry in assessing bone quality in this specific group of children. We measured outcome of bone quality and, because these children tend to have altered skeletal maturation, we also studied bone age.

**Materials and methods:**

We used hand radiographs obtained in 95 children (mean age 11.4 years) presenting at outpatient paediatric clinics. We used BoneXpert software to determine bone quality, expressed as paediatric bone index and bone age.

**Results:**

Regarding feasibility, we successfully obtained a paediatric bone index in 60 children (63.2%). The results on bone quality showed a mean paediatric bone index standard deviation score of −1.85, significantly lower than that of healthy peers (*P* < 0.0001). Almost 50% of the children had severely diminished bone quality. In 64% of the children bone age diverged more than 1 year from chronological age. This mostly concerned delayed bone maturation.

**Conclusion:**

Automated radiogrammetry is feasible for evaluating bone quality in children who have disabilities but not severe contractures. Bone quality in these children is severely diminished. Because bone maturation frequently deviated from chronological age, we recommend comparison to bone-age-related reference values.

## Introduction

Children with severe neurological impairment and intellectual disability are susceptible to developing low bone mineral density, which can lead to fractures originating from a limited or even unknown trauma [1–5].

In both adults and children, bone mineral density is generally measured with dual-energy X-ray absorptiometry [3, 4, 6]. Measurement in children requires specific software and adapted reference values [7, 8]. In the Netherlands, paediatric dual-energy X-ray absorptiometry is only available at tertiary care centres, which restricts the use of dual-energy X-ray absorptiometry in this specific group of children. In addition, disrupting factors can negatively influence the reliability of dual-energy X-ray absorptiometry results; these factors include contractures, scoliosis and movement during measurement, which are common in this group of children [9]. Therefore it is important in this group of children at risk for low bone mineral density to find a reliable diagnostic method of screening bone quality that is generally available at hospitals, easy to apply and less prone to errors caused by disrupting factors than dual-energy X-ray absorptiometry.

With automated radiogrammetry of plain hand radiographs, both bone quality and bone age can be measured with web-based software [10]. Bone quality measured with automated radiogrammetry is expressed as paediatric bone index. This paediatric bone index is determined by geometrical calculations, similar to the determination of digital X-ray radiogrammetry bone mineral density. The digital X-ray radiogrammetry bone mineral density has been shown to correlate well with peripheral dual-energy X-ray absorptiometry measurements in studies of the forearm, the femoral neck in adults, and the lumbar spine and total body in children [10–13]. Paediatric bone index reference values have been developed in a large group of healthy children (*n* = 2,398) [10]. These reference values are related to gender and bone age [10]. In children treated for acute lymphoblastic leukaemia and growth hormone deficiency, automated radiogrammetry has been shown to be easy to apply using a negligible effective radiation dose [13]. However, no data have been published on the use of this method in children with severe neurological impairment and intellectual disability.

Bone age can be determined automatically based on either the Greulich and Pyle or the Tanner and Whitehouse reference [14]. Age-related reference values are generally used in diagnostic methods (such as dual-energy X-ray absorptiometry and quantitative ultrasound [US]) used to assess bone quality in children [15].

In clinical practice, severely disabled children frequently have compromised growth velocity, whereas their skeletal maturation can be either delayed or accelerated [2, 16–18]. This evokes the question whether a difference exists between bone age and chronological age in children with severe neurological impairment and intellectual disability. Therefore the aims of this study were to determine the feasibility of automated radiogrammetry in children with severe neurological impairment and intellectual disability, and to obtain results on bone quality and bone age in this group of children. Additionally, we assessed differences between bone age and chronological age in this group of severely disabled children.

## Materials and methods

### Study design

This study was part of a cross-sectional multicentre study on bone quality in children with severe neurological impairment and intellectual disability. Four Dutch hospitals participated. Together these four hospitals cover a large part of the southwest of the Netherlands. The ethics committees of the Erasmus University Medical Center Rotterdam (MEC-2005-182) and of each participating hospital approved this study.

### Study population

We included children with severe neurological impairment and intellectual disability. These children were known to have a moderate or severe intellectual disability (intelligence quotient < 55) and a gross motor functioning classification system level IV or V; all children visited the outpatient paediatric clinic of a participating hospital. The five-level Gross Motor Function Classification System is widely used for children with cerebral palsy and describes gross motor function on the basis of self-initiated movement [19]. Children in level IV may walk short distances with physical assistance of an adult at home but use wheeled mobility outdoors. Children in level V always use a wheelchair for mobility and have severe limitations in head and trunk control [19, 20].

Children were subdivided into five groups according to the aetiology of their disability. The first group consisted of children with a congenital cause, for example lissencephaly; the second group had perinatal complications, for example placental abruption; the third group had acquired disabilities like meningitis or trauma; the fourth group included children with a combination of congenital and acquired causes; the final group included children with an unknown cause of disability. Ninety-five children were included from June 2006 until January 2009.

### Automated radiogrammetry

Automated radiogrammetry analysis was usually performed on a posterior anterior radiograph of the non-dominant hand. In cases where fewer contractures were present in the right hand, the right hand was used. To get optimal radiographic results, one of the authors (S.M., with 10 years of experience in Intellectual Disability Medicine (ID-medicine)) was present during all radiographic examinations. A maximum of two radiographs were taken. The preferred method was digital radiography in DICOM format, but traditional film radiographs could also be used once digitised using the Diagnostic Pro Advantage Scanner (VIDAR Systems Corp., Herndon, VA).

**Fig. 1:**
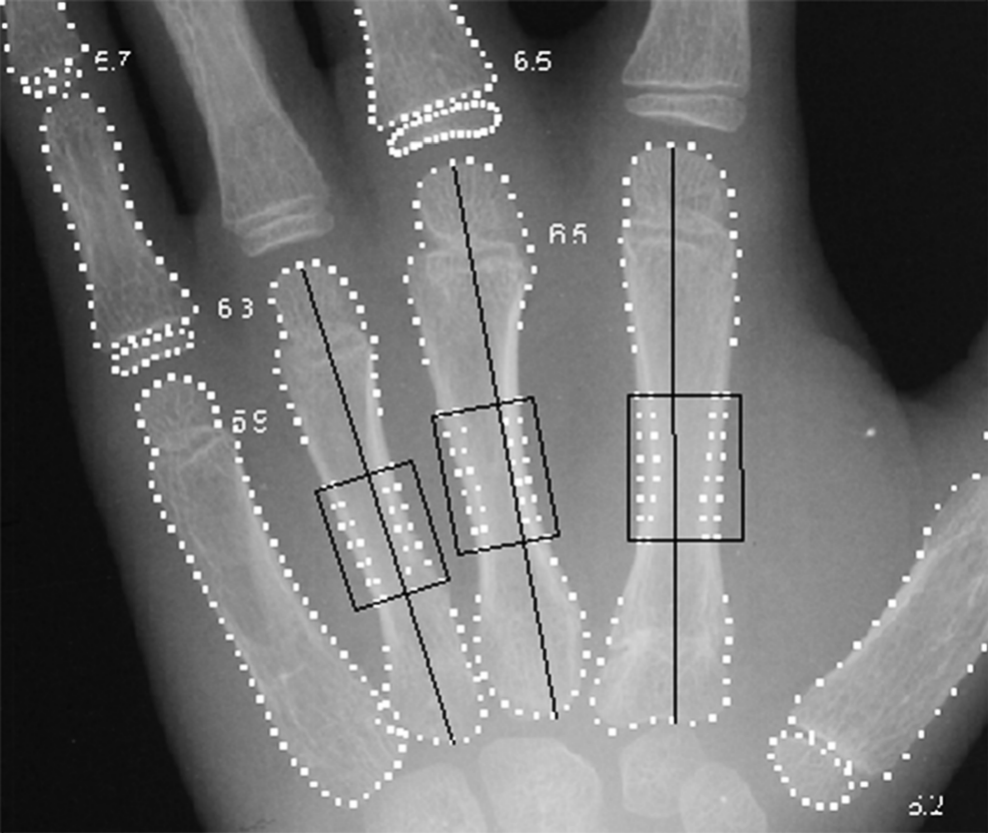
Bone age. Hand radiograph of a 6-year-old girl shows the bone borders used for calculating bone age (*dotted white lines* around the individual bones, or ossification centres) and paediatric bone index (*black boxes* in metacarpals II through IV), as outlined by BoneXpert. The small numbers represent the given bone age for the individual bones used in calculating bone age

Automated radiogrammetry was performed with BoneXpert© (software version 1.14; Visiana, Holte, Denmark). This method determines the bone ages of the middle three metacarpals and a minimum of eight other hand bones (Fig. 1).

### Feasibility

Feasibility was specified in terms of successful determination of paediatric bone index. The authors considered the method feasible if the paediatric bone index standard deviation score could be obtained in at least 70% of the children.

### Bone quality

Paediatric bone index was calculated using the three middle metacarpals by a formula containing the average values for transverse cortical area (A), bone width (W) and bone length (L): paediatric bone index = A/(W^1.33^ L^0.33^) [10]. Individual paediatric bone index outcomes were compared to reference values determined in healthy bone age- and gender- matched children and expressed in a standard deviation score [10].

### Bone age

BoneXpert can automatically determine either the Greulich and Pyle bone age or the Tanner and Whitehouse bone age. In this study we used the Greulich and Pyle reference, which has been found to be a robust method of automatic determination of bone age [14]. Valid and consistent bone ages of at least eight bones were required to assess bone age [21]. The difference between chronological age and bone age was calculated as automated bone age (years) minus chronological age (years). Based on clinical experience, a difference of 1 year or more in either direction was defined as relevant.

### Statistical analysis

Statistical analysis was performed using Statistical Package for Social Sciences for Windows 15.0 (IBM, Armonk, NY). Results were expressed as mean ± standard deviation. We used Student’s *t*-tests and Pearson chi-square tests to calculate differences between groups (children with a measurable paediatric bone index standard deviation score versus children with no measurable paediatric bone index standard deviation score). A *P*-value of less than 0.05 was considered statistically significant.

## Results

Patient characteristics are summarized in Table 1. We included 95 children with a mean age of 11.4 years (standard deviation [SD] 4.8 years); 53 (55.8%) were male. Eighty percent of the children had a gross motor functioning classification level V. The most common causes of disability were congenital (40%) and perinatal (31%). Mean weight of the children was 32.3 kilograms (range 19.7–44.7 kg), and 82.1% of the children had epilepsy.

**Table 1:** Characteristics of the study population (*n* = 95)

Age^a^ in years		11.4 (4.8)
Weight^a^ in kilograms		32.3 (12.5)
Gender	Male/female	53/42
Epilepsy		78
Gross motor functioning classification system	Level IV/V	19/76
Aetiology of disability	Congenital Perinatal Acquired Combination of congenital and acquired Unknown	38 29 7 4 17

^a^mean with standard deviation in parentheses

### Feasibility

**Fig. 2:**
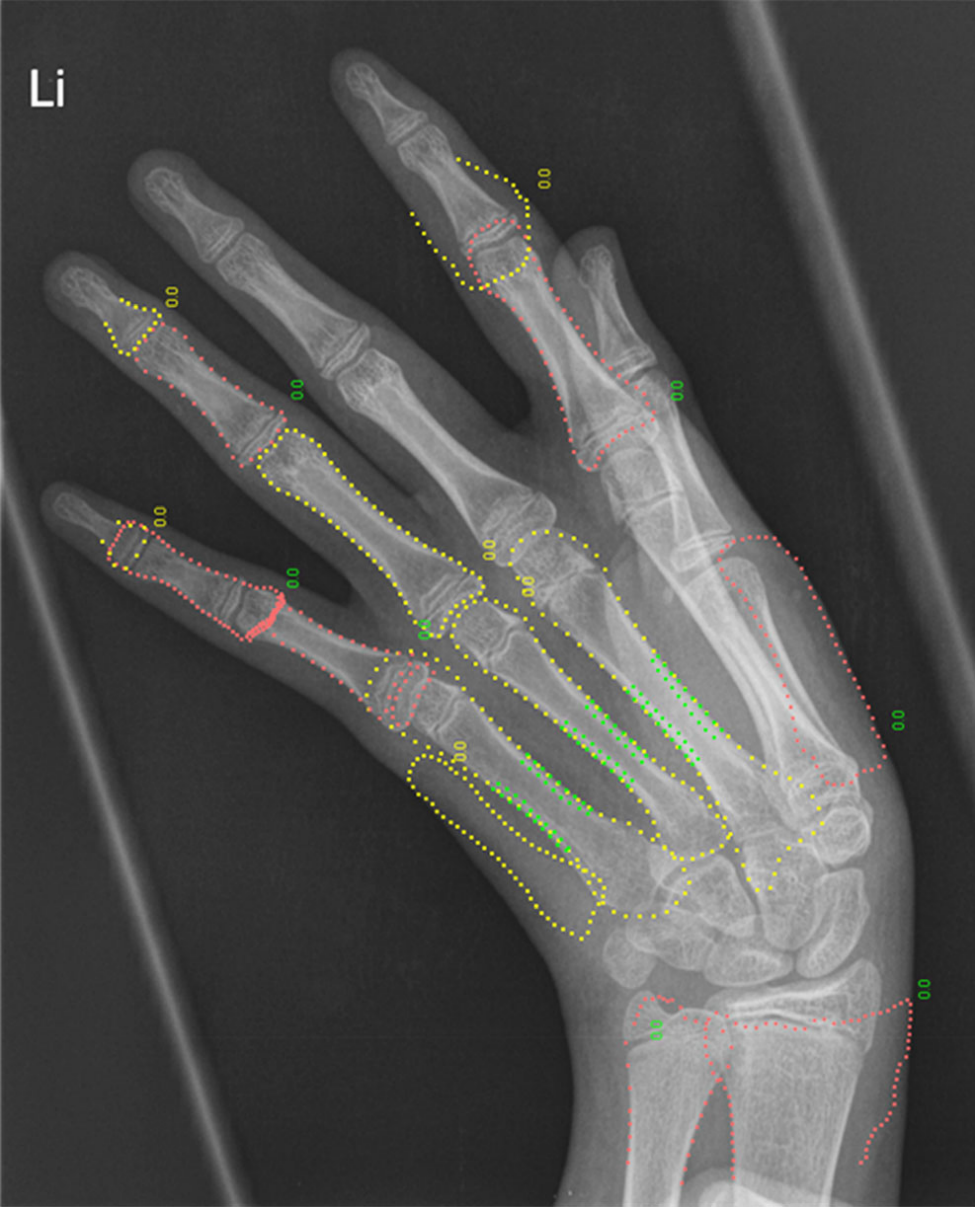
Failed bone age determination using a hand radiograph in a 13-year-old girl with projection of the metacarpals. Hand contractures causing overprojection of the metacarpals prevented BoneXpert software from obtaining the correct positioning of the dotted lines on the bone edges and correct positioning of the regions of interest in this case. *Li* left

Hand radiographs were obtained from all 95 children. The paediatric bone index standard deviation score could be calculated from 60 radiographs (63.2%; 95% confidence interval 53.5–72.9%). Bone age determination was not possible in 35 children. Unsuccessful measurements had various causes (Table 2). The most common cause was contractures of the hand, which resulted in crossed projection of the metacarpals on the radiograph (Fig. 2).

**Table 2:** Reasons for failure to obtain paediatric bone index standard deviation score (*n* = 35)

Reason for failure	Number	Percentage
Missing bone age	2	5.7
Contractures of the hand causing crossed projection of the metacarpals (Fig. 2)	17	48.6
Excessive sharpening giving lack of contrast between bone tissue and surrounding soft tissue	8	22.9
Anatomical deformities of the bones (not possible to determine exact margins of regions of interest)	3	8.6
Unclear	5	14.3

Assessment of bone quality was more frequently unsuccessful in children with more severe motor disabilities, scored as gross motor functioning classification system level V (chi-square test, *P* = 0.03). Age, gender, aetiology of the disability, weight and epilepsy did not influence feasibility.

### Bone quality

The mean paediatric bone index standard deviation score in these 60 children was −1.85 (SD 1.9), significantly lower than that of healthy peers with the same bone age (*P* < 0.0001). There was no difference in mean paediatric bone index standard deviation score between boys and girls (*P* = 0.35). Paediatric bone index standard deviation score was not associated with age (*P* = 0.84). In 29/60 children (48.3%) the paediatric bone index standard deviation score was less than −2.0.

### Bone age

**Fig. 3:**
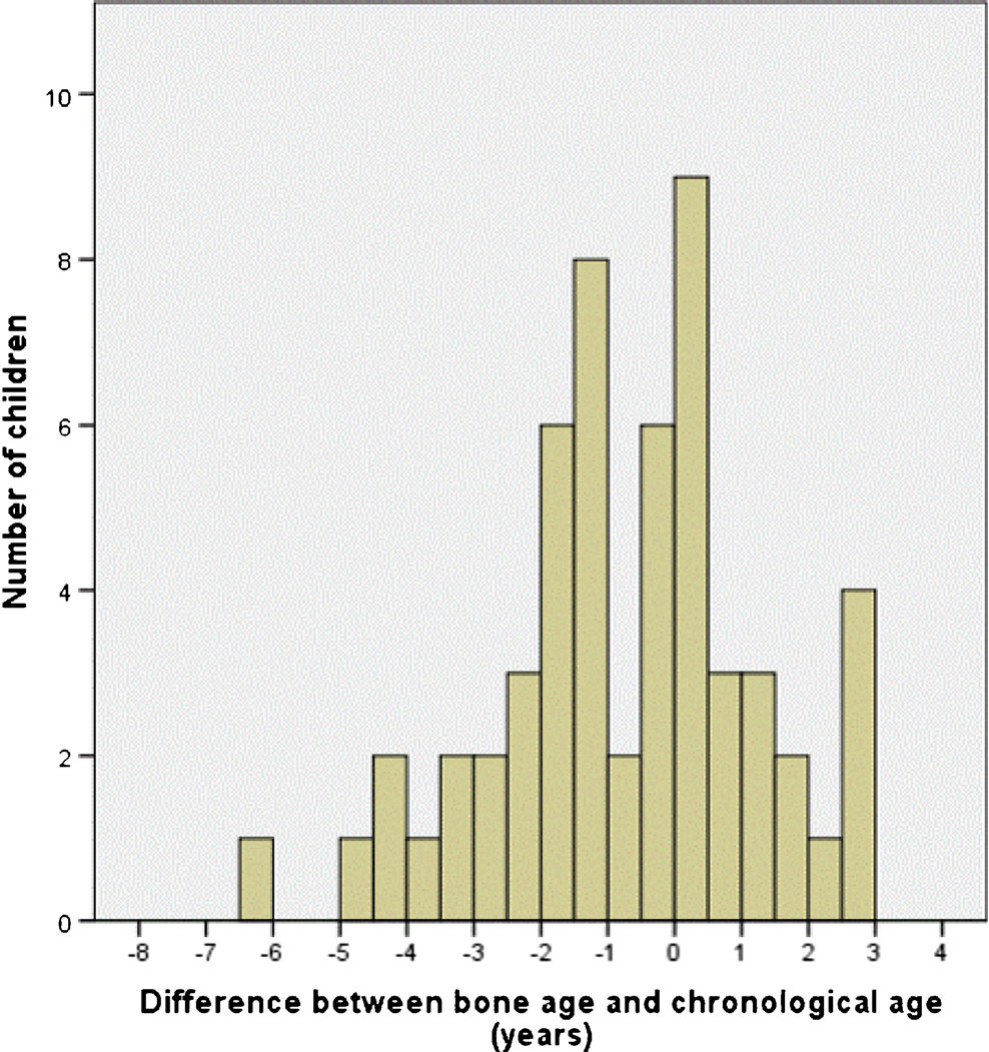
Histogram shows differences between chronological age and bone age (*n* = 56). Chronological age is defined as age in years at time of measurement. Bone age is the automated bone age in years as determined by BoneXpert software based on hand radiographs

To determine bone age successfully, images of at least eight hand and wrist bones were required. Bone age could not be measured in four children. Bone age and chronological age diverged more than 1 year in 36/56 (64%; 95% CI 51.7–76.8%) children. Bone maturation was delayed in 26 children and accelerated in 10. Individual differences between both values varied from a bone age 3 years ahead of chronological age to 6 years behind (Fig. 3).

## Discussion

This is a unique study using automated radiogrammetry in children with severe neurological impairment and intellectual disability. We assessed feasibility of using radiogrammetry to determine bone quality and bone age in this severely disabled group. We successfully determined bone index standard deviation score in 60 of 95 (63.2%) children with severe neurological impairment and intellectual disability. This percentage is slightly lower than the minimum of 70% we required for the method to be considered feasible for this group of children. Bone quality determination was more difficult in children with a gross motor functioning classification system level V. These children have severe contractures, which was the most common cause for unsuccessful measurement (17/35, 49%). This made correct identification of bone edges, necessary for automated assessment of the paediatric bone index, impossible. Although this reduces the usability of the automated radiogrammetry method in the most severely disabled paediatric group, it is important to realise that in children with severe contractures and deviant posture other diagnostic methods are also difficult to apply [9, 22].

The mean paediatric bone index standard deviation score in this group of children was −1.84 (SD: 1.93) and almost half of the children had a paediatric bone index standard deviation score lower than −2, demonstrating that bone quality is clearly diminished in children with severe neurological impairment and intellectual disability in comparison to healthy children with similar bone age and gender [23]. Measurements failed more often in children with more severe motor disability. Motor dysfunction is an important risk factor of low bone density [23]. Therefore at least some of the children in whom the measurement failed could have been diagnosed with (very) low paediatric bone index. Accordingly, the actual frequency of low bone density in the overall group may be even higher.

The BoneXpert method uses automated bone age determination, preventing interobserver variation. Bone age and chronological age were found to diverge in a substantial number of children; the difference was more than 1 year in 36/56 children (64.3%). Comparing the paediatric bone index outcome value to bone-age-related reference values appears to result in more accurate outcome measures, because skeletal growth and maturation, and bone mineral accrual appear to be closely related [24]. These important aspects of growth in children with severe neurological impairment and intellectual disability might similarly hinder other diagnostic methods of bone mineral density and bone quality [15].

Children with severe neurological impairment and intellectual disability compose a small but vulnerable group of patients requiring frequent medical care and interventions at hospitals and other care centres. Therefore study of these children is limited by the small number of patients in this group. However, fractures associated with compromised bone health are frequently present in this group. In addition to an increasing life expectancy in these children resulting from improved health care (for example by introduction of the gastrostomy catheters that improve nutritional state), an increasing fracture incidence can be expected. Therefore automated radiogrammetry might be helpful in the long-term evaluation of bone quality in this group.

## Conclusion

Automated radiogrammetry is a feasible method to determine paediatric bone index in children with severe neurological impairment and intellectual disability but without severe contractures. Bone quality is clearly diminished in this group of children in comparison to healthy children with the same bone age and gender. Also, bone age and chronological age were found to diverge in a substantial number of the children. Bone age should be taken into account when other diagnostic methods (for example dual-energy X-ray absorptiometry and quantitative ultrasound) are used to determine bone quality in this group.
